# Our Experience of Trauma Management During Novel Coronovirus 2019 (COVID-19) Pandemic in a Busy Trauma Center in Southern Iran

**DOI:** 10.30476/BEAT.2020.87029

**Published:** 2020-07

**Authors:** Hossein Akbarialiabad, Hossein Abdolrahimzadeh Fard, Hamid Reza Abbasi, Shahram Bolandparvaz, Shahin Mohseni, Vahid Mehrnous, Mina Saleh, Sima Roushenas, Shahram Paydar

**Affiliations:** 1 *Student Research Committee, Shiraz medical school, Shiraz, Iran*; 2 *Trauma Research Center, Shahid Rajaee (Emtiaz) Trauma Hospital, Shiraz University of Medical Sciences, Shiraz, Iran*; 3 *Department of Surgery, Orebro University Hospital, Orebro, Sweden*; 4 *Section of Trauma, Acute Care, and Global Surgery, Division of general surgery, Department of Surgery, University of British Columbia*; 5 *MSc in surgery student, Department of Surgery, University of British Columbia*

**Keywords:** COVID-19, Trauma, Surgery, Pandemics, Communicable disease control

## Abstract

During the past few months, the novel coronavirus 2019 (COVID-19) pandemic has significantly affected medical service provision. In Iran, it has caused around 197,000 inflictions and 9200 deaths up to June 18, 2020. While many departments turned to telehealth in this era, the trauma service should provide non-stop in presence service to the trauma victims. Our trauma center is the largest in the southwest of Iran, with the mean annual admission of 18,500 polytrauma patients. In this center, we designed a safety protocol to mitigate the spread of disease and also have a more robust case finding system, especially among asymptomatic carriers who attend hospitals based on their trauma. In brief, all unstable patients were considered SARS-COV-2 positive and were directed toward the Specialized COVID-19 related ICU. For all stable patients, history, physical examination, CXR, and lab test (Complete Blood Count, Erythrocyte Sedimentation Rate, C-Reactive Protein) were ordered before entering the wards. If there was any suspicion of COVID-19, the stable patient was admitted to the COVID-19 specialized ward. Among all 1805 patients admitted during a ten weeks interval (from January 30, 2020, to April 14, 2020), 84 had a red flag and toward to COVID-19 related wards. Of those, 67 had positive PCR or evidence in CT in favor of the COOVID-19. Moreover, during regular workups, we found that 19 completely asymptomatic trauma victims had typical Chest CT scan findings of COVID-19.

## Introduction

Likewise, in several countries around the world, Iran encountered a significant health challenge during the past few months. Up to June 18, 2020, the novel coronavirus 2019 (COVID-19) pandemic has resulted in, officially declared, 197,000 inflictions with around 9200 deaths [1]. In this catastrophic era, and parallel to mass quarantine, telehealth has prevailed among many specialties to reduce the necessity of presence in clinical settings [2, 3]. Tertiary trauma hospitals, however, have to provide related services. It should be noted that it is the largest trauma center in the south of Iran with annual, on average, 18,500 polytrauma admissions. In this retrospective study, we investigated entrance to this hospital over ten weeks starting from January 30, 2020, to April 14, 2020. 

During this period, a total of 4167 trauma victims were attended or referred to the hospital. The frequency of trauma for each type is available in Table 1. As it is clear, the fall (from less than 3 feet) and MVA (motor vehicle accident) were the major causes with 23.5% and 21.9%, respectively. On the contrary, self-inflicted injuries and penetrating assaults were the least causal factors with 1.5% and 3.3%, respectively. From these 4167, 1805 were planned to be admitted or being observed beyond 6 hours. During the COVID-19 period, in Shiraz Trauma Hospital, accordingly, a safety protocol was developed and implemented to mitigate the risk of COVID-19 transmission among trauma health care professionals and patients. 

This algorithm was used to whom the plan was admission or observation for more than 6 hours. On arrival, the patients were considered “probable” COVID positive cases. Unstable patients were transferred to an ICU dedicated to critically ill COVID-19 patients, and further confirmative workups (mainly CT scans) were performed. Stable patients had a complete physical examination and history taking, while related universal precautions were strictly followed. If any signs/symptoms, history, lab data, and imaging were contributory to COVID-19 diagnosis, they were transferred to specialized COVID-19 wards, and PCR or/and Chest CT scan (preferably), were obtained to confirm the diagnosis. Since access to PCR tests was limited, all stable patients performed a chest x-ray and had their blood sampled for a complete blood count, CRP, and erythrocyte sedimentation rate (ESR). 

Of 1,805 Patients, 84 (5%), based on symptoms or a positive history, abnormal laboratory/imaging, considered “suspected” cases, and 67 (4%) were “confirmed” cases by the confirmatory tests. Given that a considerable amount of multiple trauma patients need chest and abdominal CT scans, some incidental findings highly raised suspicion of COVID-19 that 19 (1%) utterly asymptomatic patients with clear history and negative lab/imaging results for COVID-19 had the typical radiological findings of COVID-19 on their chest CT scan. 

We discerned a notable prevalence of carriers/infected individuals among trauma patients. Although our study is based on a single-center experience, we suppose that in high prevalent areas of COVID-19, all patients, even with unrelated chief complaints, should be considered positive unless proven otherwise. As mentioned by one study, the true prevalence of the disease can be more due to low negative predictive value and diversity in terms of the accuracy of PCR tests [4]. We highly suggest full protection for emergency care staff and universal testing of patients, if possible. Contamination of uninfected trauma victims may exacerbate their already tenous conditions.

**Table 1:** Frequency and mode of trauma to all patients attended the Rajaee (Emtiaz) Trauma Hospital from January 30, 2020, to April 14, 2020

	Penetrating assault	Non-penetrating assault	MVA	Bike accident	Pedestrian accident	High drop (˃3 feet)	Fall (˂3 feet)	Occupational or home injuries (non-assault home injuries)	Self-injury (suicide)	Other	Total
Number	137	624	708	513	296	272	829	619	63	106	4167
Percent	3.3%	15%	17%	12.3%	7.1%	6.5%	19.9%	14.9%	1.5%	2.5%	100%

**Fig. 1:**
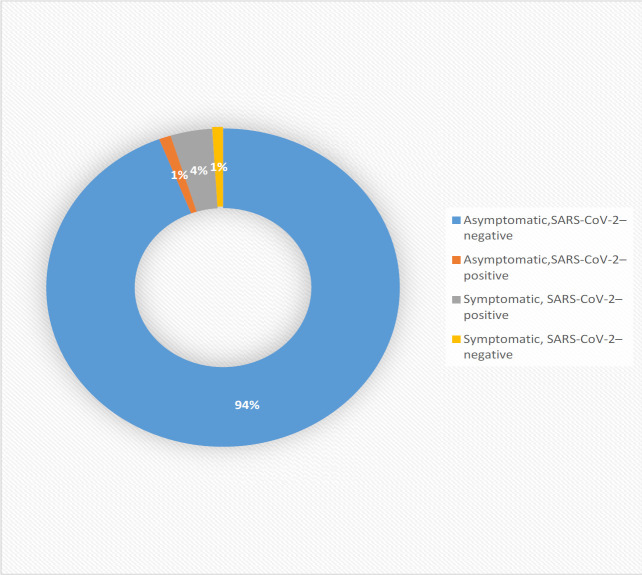
Symptom Status and SARS-CoV-2 test Results among 1805 traumatic patients admitted in Rajaee (Emtiaz) Trauma Hospital

## Conflict of Interest:

None.
